# Attention Code Blue: a comprehension of in-hospital cardiac arrest from a multispeciality hospital in South India

**DOI:** 10.1186/cc14492

**Published:** 2015-03-16

**Authors:** M Hisham, MN Sivakumar, T Sureshkumar, R Senthil Kumar, A Satheesh

**Affiliations:** 1 Kovai Medical Center and Hospital, Coimbatore, India

## Introduction

Numerous American and European studies have associated survival rates of in-hospital cardiac arrest (IHCA) with different quality markers. There has been a paucity of studies that explain IHCA in Asian populations. This study was conducted to assess the characteristics and survival among patients suffering from IHCA.

## Methods

All Code Blue activations from 1 January 2012 to 31 December 2012 were analyzed retrospectively. Data were gathered from the Code Blue form and finer details of individual patients were linked through their medical records. Code Blue was activated only for events that happened outside the medical and surgical ICUs.

## Results

A total of 260 Code Blue activations were made, out of which there were 203 true cardiac arrest events among 40,168 in-patients; the cumulative incidence of the same was 0.51%. Mean (SD) duration of arrival of the Code Blue Team (CBT) to the scene was 64.5 (27.7) seconds. Cardiovascular illness was the predominant baseline morbidity but none of the baseline illness showed increased risk of mortality in this group. Among true cardiac arrest events, 92.6% was due to pulseless electrical activity (PEA)/asystole and 7.7% was due to ventricular fibrillation (VF)/pulseless ventricular tachycardia (VT); both of these did not have any difference on the initial outcome. But having an initial rhythm of VF/pulseless VT had 90% more chance for discharge from the hospital, with *P *= 0.04. Although arrival time of the CBT did not have any influence on the final outcome, duration of resuscitation ≤20 minutes had an odds ratio of 10.6 with *P *< 0.001 favoring return of spontaneous circulation over death after controlling for age. Of the 203 patients who had true cardiac arrest events, 43 (21.2%) were discharged from the hospital. Good neurological outcome at discharge was seen among 22 (10.8%) of the patients based on Cerebral Performance Category Score.

## Conclusion

Our experience shows that out of every 1,000 patients admitted to our hospital, about five sustained cardiac arrest, of whom only 11.3% survived to hospital discharge with good neurological recovery. Variation in the effectiveness of the cardiopulmonary resuscitation quality in comparison with world data could be due to the inherent difference in the severity of the primary illness in the patients and diversity in the reported data.
